# Adventitial Vessel Growth and Progenitor Cells Activation in an *Ex Vivo* Culture System Mimicking Human Saphenous Vein Wall Strain after Coronary Artery Bypass Grafting

**DOI:** 10.1371/journal.pone.0117409

**Published:** 2015-02-17

**Authors:** Francesca Prandi, Marco Piola, Monica Soncini, Claudia Colussi, Yuri D’Alessandra, Eleonora Penza, Marco Agrifoglio, Maria Cristina Vinci, Gianluca Polvani, Carlo Gaetano, Gianfranco Beniamino Fiore, Maurizio Pesce

**Affiliations:** 1 Unità di Ingegneria Tissutale, Centro Cardiologico Monzino, IRCCS, Milan, Italy; 2 Politecnico di Milano, Dipartimento di Elettronica, Informazione e Bioingegneria, Milan, Italy; 3 Istituto di Patologia Medica, Università Cattolica del Sacro Cuore, Rome, Italy; 4 Unità di Immunologia e Genomica Funzionale, Centro Cardiologico Monzino, IRCCS, Milan, Italy; 5 II Divisione di Cardiochirurgia, Centro Cardiologico Monzino, IRCCS, Milan, Italy; 6 Dipartimento di Scienze Cliniche e di Comunità, Università di Milano, Milan, Italy; 7 Division of Cardiovascular Epigenetics, Goethe University, Frankfurt-am-Main, Germany; Bristol Heart Institute, University of Bristol, UNITED KINGDOM

## Abstract

Saphenous vein graft disease is a timely problem in coronary artery bypass grafting. Indeed, after exposure of the vein to arterial blood flow, a progressive modification in the wall begins, due to proliferation of smooth muscle cells in the intima. As a consequence, the graft progressively occludes and this leads to recurrent ischemia. In the present study we employed a novel *ex vivo* culture system to assess the biological effects of arterial-like pressure on the human saphenous vein structure and physiology, and to compare the results to those achieved in the presence of a constant low pressure and flow mimicking the physiologic vein perfusion. While under both conditions we found an activation of Matrix Metallo-Proteases 2/9 and of microRNAs-21/146a/221, a specific effect of the arterial-like pressure was observed. This consisted in a marked geometrical remodeling, in the suppression of Tissue Inhibitor of Metallo-Protease-1, in the enhanced expression of *TGF-β_1_* and *BMP-2* mRNAs and, finally, in the upregulation of microRNAs-138/200b/200c. In addition, the veins exposed to arterial-like pressure showed an increase in the density of the adventitial *vasa vasorum* and of cells co-expressing NG2, CD44 and SM22α markers in the adventitia. Cells with nuclear expression of Sox-10, a transcription factor characterizing multipotent vascular stem cells, were finally found in adventitial vessels. Our findings suggest, for the first time, a role of arterial-like wall strain in the activation of *pro*-pathologic pathways resulting in adventitial vessels growth, activation of *vasa vasorum *cells, and upregulation of specific gene products associated to vascular remodeling and inflammation.

## Introduction

Coronary artery bypass grafting (CABG) is a surgical procedure routinely used to re-vascularize the chronically ischemic myocardium since ‘60s [1]. Despite the immediate benefits resulting from restoration of the correct myocardial perfusion, patients receiving saphenous vein (SV) bypass undergo mid- and long-term complications caused by progressive patency reduction [2,3]. While vascular conduits derived from arterial sources such as the inner mammary or the radial arteries are preferred for their lower propensity to stenosis [4], the employment of the SV is unavoidable, especially in cases of ‘multi-vessel’ pathology[5]. In these instances, even if “no touch” SV harvesting modalities preserving the vascular integrity have been introduced [4], there is still a high incidence of venous bypass failure. Vein bypass stenosis is caused by an overgrowth of smooth muscle cells (SMCs). These cells, switching from a contractile to a migratory/secretory phenotype [3,6], invade the intima and narrow the vessel lumen. Secondary effects such as atherosclerosis have been also reported [3]. Finally, activation and recruitment of vein-resident cells with mesenchymal progenitor characteristics has been hypothesized [7–10].

Numerous studies have addressed the pathophysiology of vein graft disease. These experimental models, performed by transplanting autologous vein segments into arterial position in animal models [11] or by culturing human vein segments using standard tissue culture techniques, have led to understand the contribution of different cellular species to intima hyperplasia [12–14], to assess the phenotypic changes occurring in vein cells during arterialization[15], to test intervention strategies with gene transfer or gene modulation approaches [16] and, finally to identify novel molecular pathways based on micro-RNAs-dependent gene expression programs [17,18].

A relevance of the altered hemodynamics in venous grafts failure has been also hypothesized. This is based, for example on the finding that endothelial cells and smooth muscle cells respond to shear stress and cyclic strain with apoptosis [19], modified proliferation [20], enhanced or reduced migratory activity [21], as well as with a modification in redox state and cytokine synthesis [22], and that early structural adaptation of the vein to the arterial flow, as detected, e.g., by CT-scan- or 3D-Echo-derived imaging data [23,24], predicts the temporal evolution of the graft patency in patients [25].

In the present contribution we used an *ex vivo* culture system allowing to stimulate human SV segments with a low pressure consistent with the venous circulation, or arterial-like pressure [26], to investigate the biomechanical effects of the arterialization on expression of vascular pathology targets.

## Results

### Strain-dependent remodeling of the human SV

**Fig 1:**
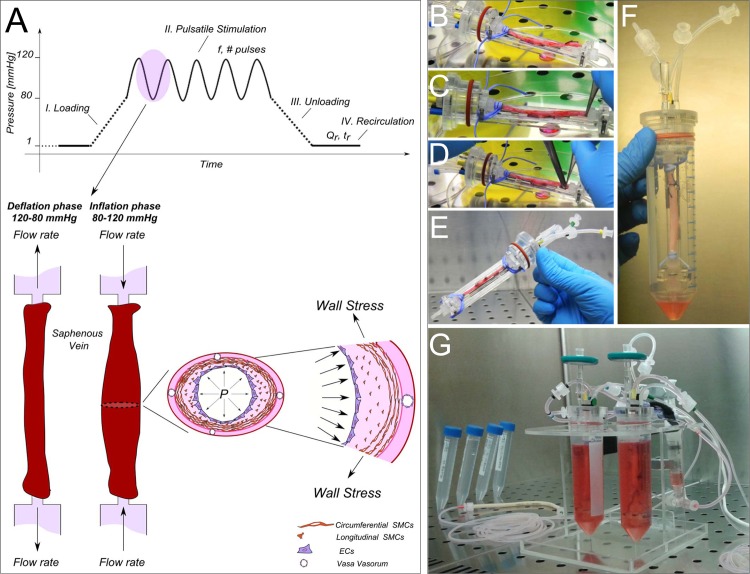
The compact and automated *ex vivo* vessel culture system able to artificially produce the effects of the arterial pressure-related cyclic wall distention. (A) The single pressure stimulation cycle consists of: *i)* a loading phase (the luminal pressure reaches 80 mmHg); *ii)* a pulsatile stimulation phase (pressure oscillates between 80–120 mmHg at a desired pulse rate); *iii)* an unloading phase (pressure is lowered to zero); and *iv)* a recirculation phase with a constant flow rate allowing a metabolic supply to the vessel. All the specific parameters can be set through the software interface; namely: pressure values, the pulse frequency (*f*)/number of cycles (*# pulses*) for the stimulation period, the duration (*t*_*r*_) and the medium flow rate (*Q*_*r*_) of the recirculation period. During the inflation phase the cells covering the lumen (ECs) and those embedded in the medial layer (SMCs) are subjected to circumferential stress and strain typical of the arterial circulation. (B-E) The *ex vivo* vessel culture system during assembling under laminar flow hood. The SV segments are cannulated on both ends with barbed luer fittings, and bound to the connectors using a vessel loop. The SV housing is then inserted within the 50-ml tube acting as reservoir (F). Once assembled, the culture chamber is connected to a stimulation circuit (G).

The SV samples were mounted into an *ex vivo* culture system, which was designed to reproduce the physiologic venous perfusion with constant low pressure and flow, or to mimic the arterial-like stimulation with wall strain conditions typical of the coronary circulation [26] (Fig. 1). This latter condition is acted in 4 independent phases: *i)* a loading step, *ii)* a pulsatile stimulation step, *iii)* an unloading step, and *iv)* a recirculation step. While the first three phases (duration 10 min) allow a cyclical loading/unloading of the vessel with the culture medium and its stimulation with arterial-like pressure, the fourth phase (duration 2 min) was introduced to replace the medium inside the vein with the excess medium contained in the external compartment, thus maintaining a stable nutrients and oxygen supply to both sides of the vessel for the entire stimulation period.

The pressure stimulation employed in the present study has been already detailed in a previous report [26]. This (S1 Video), consisted in a circumferential strain applied to the SV wall, and in particular to the luminal endothelial cells (ECs) and the medial smooth muscle cells (SMCs). The absence of a coronary-like flow allowed to assess the effects of the cyclic circumferential strain typical of the artery environment without confounding effects by the shear stress. Results of experiments performed under the arterial-like condition (hereafter defined the CABG veins group; n = 16) or under the venous perfusion regimen (hereafter defined the VP veins group, n = 16) were pairwise compared with results obtained in non-stimulated vessels (hereafter defined the Native veins group). This was possible by the modality of vessel sampling that allowed, in most of the cases, to keep part of the native tissue before beginning the stimulations.

**Fig 2:**
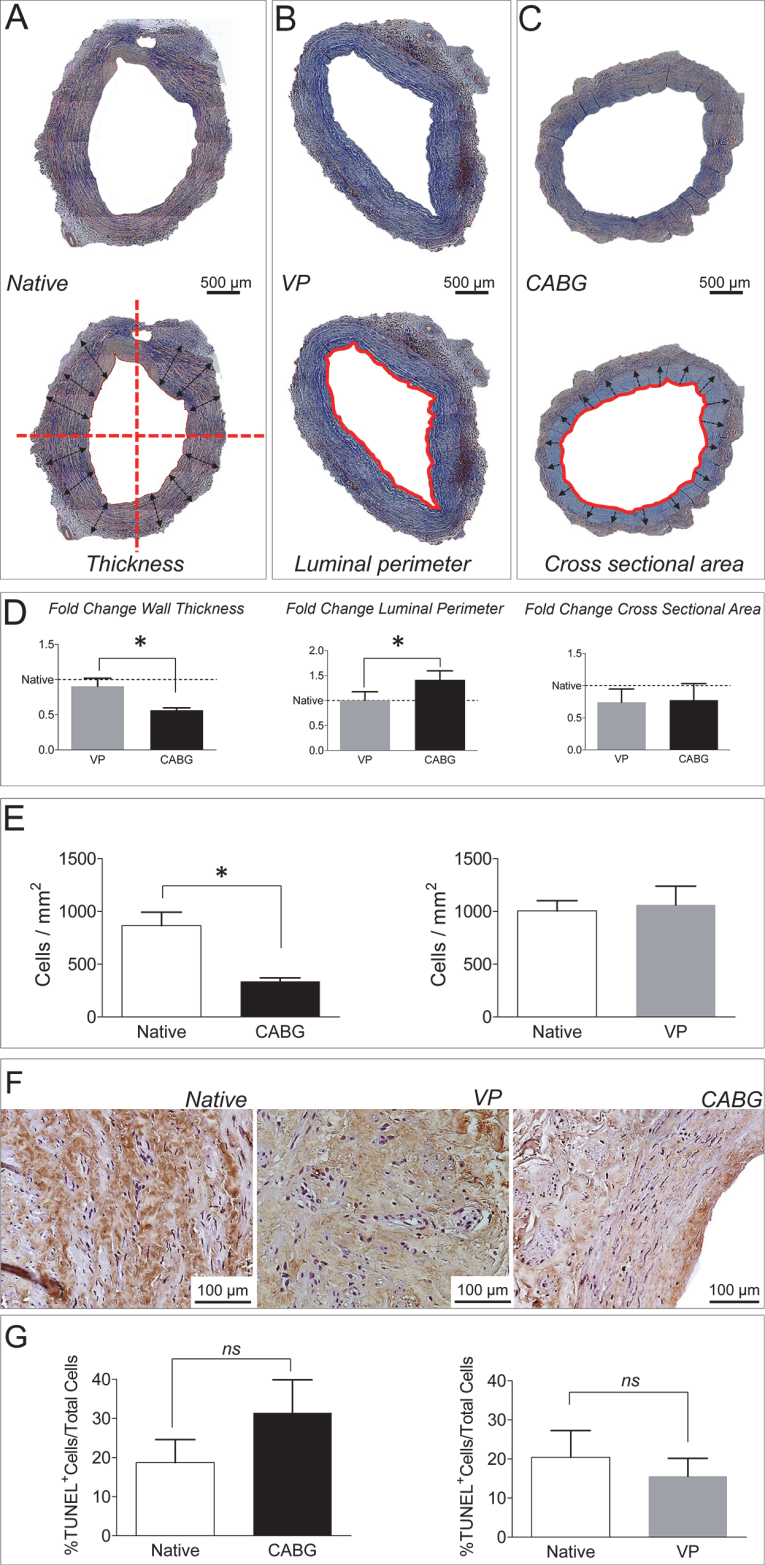
Histological appearance and morphometry of Native, VP and CABG SV samples. (A-C) Low magnification of Masson’s trichrome staining of SV transversal sections. The lower pictures in each of the panels shows, respectively an example of the morphometric estimated parameters: direction of the radius measures used to determine the wall thickness (A), the luminal perimeter (B) and the area region considered for the determination of the cross-sectional area (C). (D) Quantification of wall thickness, luminal perimeter and cross sectional area changes in VP and CABG samples vs. Native. Data are expressed as fold change vs. Native (set to 1 in the graphs); * indicates P < 0.05 by Student’s paired t-test vs. Native (n = 6). (E) Determination of cell number by nuclei counting on histological sections showing that the arterial-like pressure determines a significant reduction in the cell number. Data are shown as cell number/mm^2^. * indicates P < 0.05 by Student’s paired t-test vs. Native (n = 3). (F) Detection of TUNEL^+^ cells in Native, VP and CABG veins. (G) Quantification of TUNEL^+^ nuclei in the transversal sections of the SV samples. Statistical comparison by paired t-test (n = 3) did not show significant differences (ns).

To assess the structural changes occurring in the vein wall under the two pressure stimulation regimens, histological analysis was performed. The results showed: a decrease in the vein wall thickness, and an increase in the luminal perimeter; these changes were not associated to a modification in the tissue cross sectional area (Fig. 2 A-D). The morphological modifications determined by arterial wall strain were associated to a significant decrease in the cell density (Fig. 2E). This latter effect was, however, not the consequence of programmed cell death; in fact, CABG-treated veins did not show a significant increase in the percentage of TUNEL labeled cells (Fig. 2 F-G).

**Fig 3:**
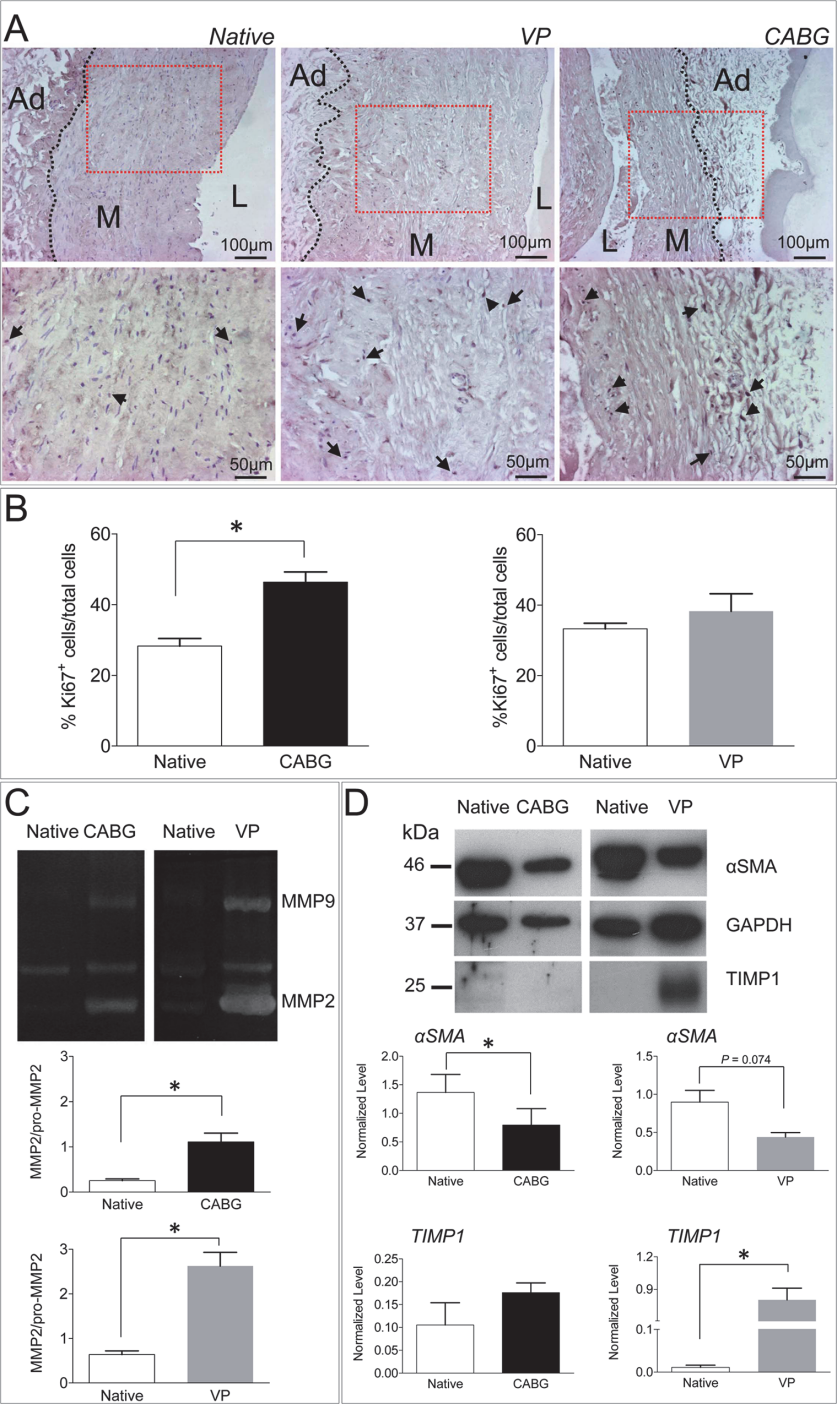
Low and high magnification of immunohistochemical staining for Ki-67 proliferation marker in Native, VP and CABG stimulated veins. Arrows indicate cell nuclei expressing Ki-67. L: lumen, M: media, Ad: adventitia. (B) Quantification of Ki-67^+^ cells, expressed as a percentage of the total in Native *vs*. CABG and Native *vs*. VP cultured SVs. * indicates *P* < 0.05 by paired t-test (n = 3). (C) Zymography of MMP-2/MMP-9 and (D) α-SMA, GAPDH and TIMP-1 Western Blot analysis in protein extracts from Native, VP and CABG stimulated SVs. The bar graphs in each panel show the quantification and statistical comparison of the MMP-2 activity (n = 5), and the α-SMA and TIMP-1 (n≥3) expression level normalized to GAPDH in Native *vs*. CABG and Native *vs*. VP samples. * indicates *P* < 0.05 by paired t-test. The *P* value above graphs is indicated in case of borderline statistical significance.

Vein arterialization is generally known to increase the proliferation of cells in the vein wall. This occurs especially in SMCs, that contribute significantly to the intima growth and the release of extracellular matrix remodeling enzymes [3]. To assess whether mechanical strain affects cells proliferation and modulates matrix remodeling, an immunolocalization of Ki-67 proliferation marker and a determination of Matrix-Metallo-Proteinases (MMPs) activity were performed in Native, VP-, and CABG-conditioned SVs by immunohistochemistry and Zymography/Western analysis, respectively. The results of immunostaining (Fig. 3A-B), showed a remarkable elevation in the percentage of Ki-67^+^ cells only in CABG samples. By contrast, the data concerning the expression of matrix remodeling enzymes indicated a similar increase in the activity and the expression of MMP-2/-9 (Fig. 3C-D) in samples treated with both pressure regimens. Interestingly, however, the Tissue Inihibitor of Matrix Metallo Proteinases (TIMP)-1 [27,28] expression was upregulated only under the venous perfusion regimen, suggesting that the lower degree of SV morphological remodeling observed in VP samples may result from a TIMP-1inhibition of the MMP activity consequent to perfusion with a physiologic pressure.

### The SV adventitia is a direct target of the wall strain, possibly predisposing to vessel restenosis

**Fig 4:**
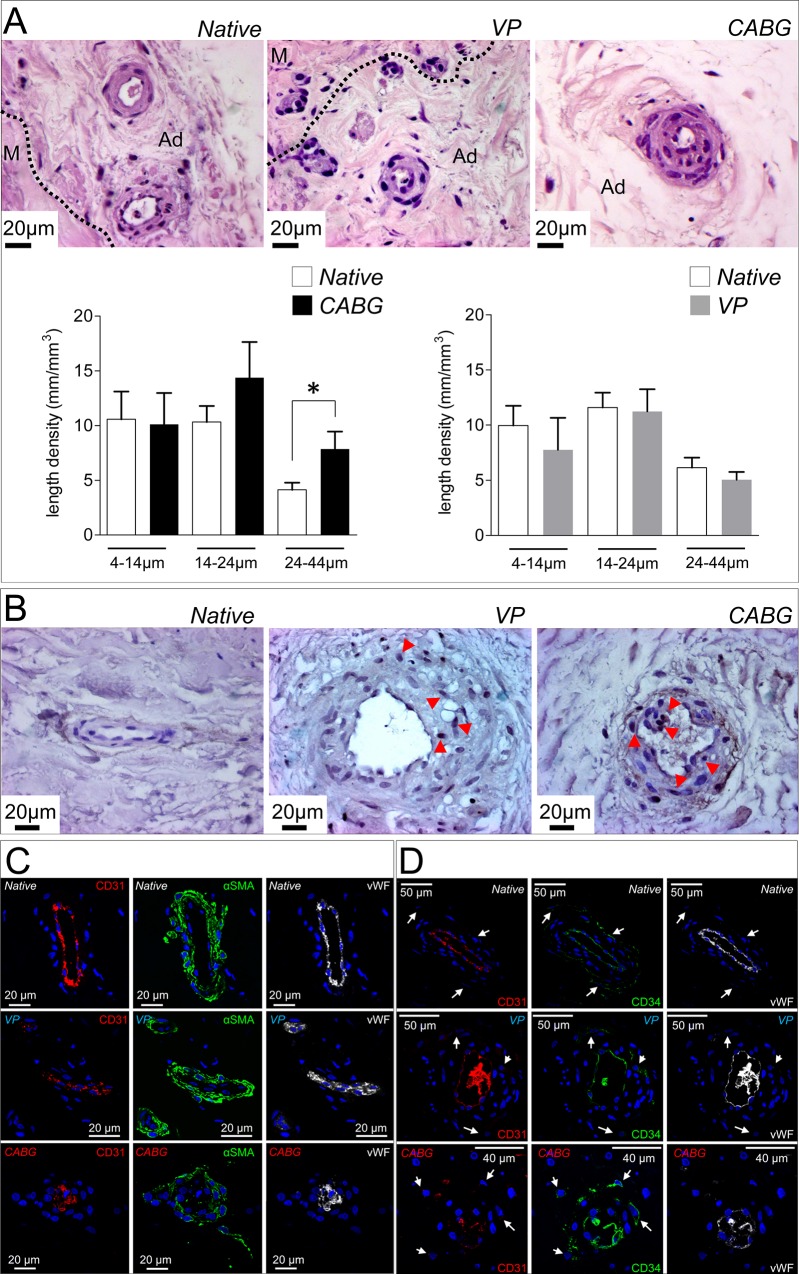
Histological appearance and marker analysis in SV *vasa vasorum*. (A) Representative micrographs of the adventitial layer in Native, VP and CABG veins stained with H&E. The boundary between the media (M) and the adventitia layers (Ad) is shown. Bar graphs indicate quantification of the *vasa vasorum* length density. The 24–44 μm category was significantly increased in CABG samples. * indicates *P* < 0.05 by paired t-test (n = 12 CABG samples; n = 12 VP samples). (B) Detection of Ki-67^+^ cells in the *vasa vasorum* of SV samples showing more abundant positive cells in adventitial vessels of CABG pressure stimulated veins (red arrowheads). Triple staining with CD31/αSMA/vWF (C) or CD31/CD34/vWF antibodies (D) to detect SMCs and ECs or SVPs in the *vasa vasorum*. An overall decrease in EC markers expression as well as α-SMA in the surrounding SMCs was observed in CABG samples. In addition, these vessels appeared remarkably de-structured.

The adventitia is a crucial regulator of vascular homeostasis [29]. Its contribution to intimal thickening has been demonstrated in animal models of vein arterialization [10,12,30,31], where invasion of adventitial cells into the medial and intimal layers has been correlated to *neo*-intima accumulation. Particular emphasis has been given to the possible contribution of the *vasa vasorum*. These accessory vessels provide nutrients to the SV wall and have been found to grow as a result of arterialization in post-mortem histological analysis of human vein bypass conduits [32]. To assess the effects of the arterial and venous pressure regimens on *vasa vasorum* structure, we then performed an accurate histological analysis of the SV adventitia. As shown in Fig. 4A, the *vasa vasorum* in CABG-stimulated veins were enlarged and surrounded by multiple cellular layers; by contrast, this change was not observed in VP samples, where *vasa vasorum* had an overall structure similar to that observed in the native veins. In order to quantitatively substantiate this finding, we determined the length density of the small (4–14μm), intermediate (14–24μm) and large (24–44μm) size adventitial vessels in transversal tissue sections [33]. As shown in bar graphs in Fig. 4A, the CABG pressure caused a significant increase in the length density of the largest size (24–44μm) *vasa vasorum*. This change was not observed in any of the VP samples *vasa vasorum* size categories, and suggested the growth of pre-existing vessels due to a specific effect of arterial-like pressure. In keeping with this hypothesis, the presence of Ki-67^+^ cells (Fig. 4B) was found in large adventitial vessels of arterial pressure-stimulated veins.

To check for possible modifications in marker expression of *vasa vasorum* cells in CABG samples, an immunofluorescence analysis was performed with antibodies to detect CD31, vWF and αSMA. Results (Fig. 4C) revealed the presence of cells with large nuclei [31] and an evident decrease in the level of SMCs and ECs markers. In particular, αSMA^+^ cells associated with *vasa vasorum* appeared to loose contact with the basal lamina and invade the surrounding adventitia (3D Z-stack reconstruction of *vasa vasorum* structure in S2 Video), suggesting that these cells might change their phenotype from contractile to secretory.

**Fig 5:**
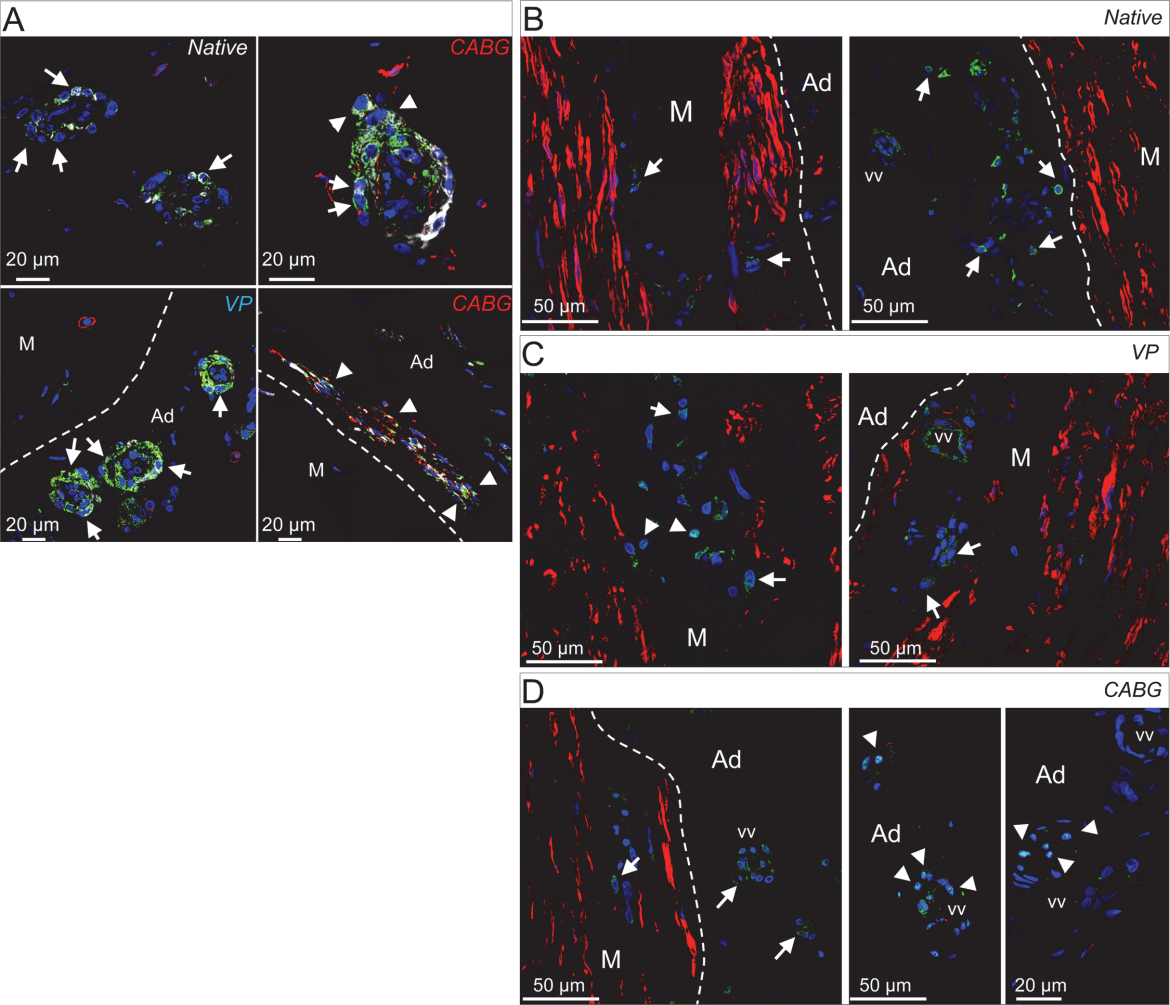
Increase in the expression of cells expressing pericyte and vascular stem cell markers in CABG pressure-stimulated SV samples. (A) Expression of NG2 (green fluorescence), SM22α (white fluorescence), and CD44 (red fluorescence). The expression of NG2 and SM22α was confined to perivascular cells located at the periphery of the *vasa vasorum* (arrows), while a higher expression of both markers was observed in both treatments with respect to Native samples. In CABG samples note the presence of cells expressing NG2 and SM22α markers de riving from *vasa vasorum* wall (upper right panel, arrowheads) and the presence of cells expressing NG2, SM22α and CD44 (arrowheads in the lower right panel) at the boundary between the adventitia (Ad) and the media (M) layers (bracketed line). (B-D) Expression of the contractile SMCs marker SM-MHC (red fluorescence) and vascular stem cells marker Sox-10 (green fluorescence) in the media and adventitia layers (arrows). In Native and VP samples cells showed Sox-10 protein in the cytoplasm, while nuclear localization of the transcription factor was found only in *vasa vasorum* in the adventitial layer of CABG- stimulated samples (arrowheads).

Participation of adventitial progenitors to vein graft disease and, more in general, to restenosis after vascular injury has been consistently shown in animal models [9,12,34–37]. Since the human SV harbors progenitor cells (the so called Saphenous Vein Progenitors, SVPs [7]) in close association with the *vasa vasorum*, we investigated the presence of these cells in veins treated with CABG or VP regimens. SVPs are characterized by CD34 and NG2, and do not express endothelial and mesenchymal markers [7]. In a first immunofluorescence staining, a CD34/CD31/vWF labeling was employed to localize SVPs in the native, VP and CABG samples. Results indicated a similar presence of undifferentiated CD34^+^/CD31^-^/vWF^-^ SVPs around the *vasa vasorum* (Fig. 4D). In a second immunostaining (Fig. 5A), the NG2 marker was analyzed in conjunction with CD44, a mesenchymal marker expressed in perivascular stem cells, by SVPs after *ex vivo* amplification, and by mesenchymal stem cells after vessel injury [7,10,38]. Staining for CD44 and NG2 was performed in conjunction with SM22α, an early marker of SMCs differentiation. Results showed that the expression of NG2 and SM22α was low in the *vasa vasorum* of the Native SV samples; by contrast they were both upregulated in the outer ring of *vasa vasorum* cells in VP and CABG conditions [7,38] (Fig. 5A). Importantly, groups of NG2^+^/SM22α^+^/CD44^+^ cells located outside the *vasa vasorum* and in proximity of the boundary between the adventitia and the media were found only in CABG samples (Fig. 5A), suggesting that cells derived from perivascular progenitors present in the adventitia were activated by a wall strain-dependent signal. The susceptibility of adventitial cells to arterial-like wall strain was finally suggested by the presence, in CABG-treated samples, of *vasa vasorum* cells showing a nuclear localization of the ‘multipotent vascular stem cells’ (MVSCs) marker Sox-10 and the absence of the mature SMC marker SM-MHC [39]. Altogether, these results indicate that adventitial cells, and in particular those associated with the *vasa vasorum*, are activated by mechanical strain in the human SV.

### Strain-dependent expression of specific miRNA and gene transcripts

**Fig 6:**
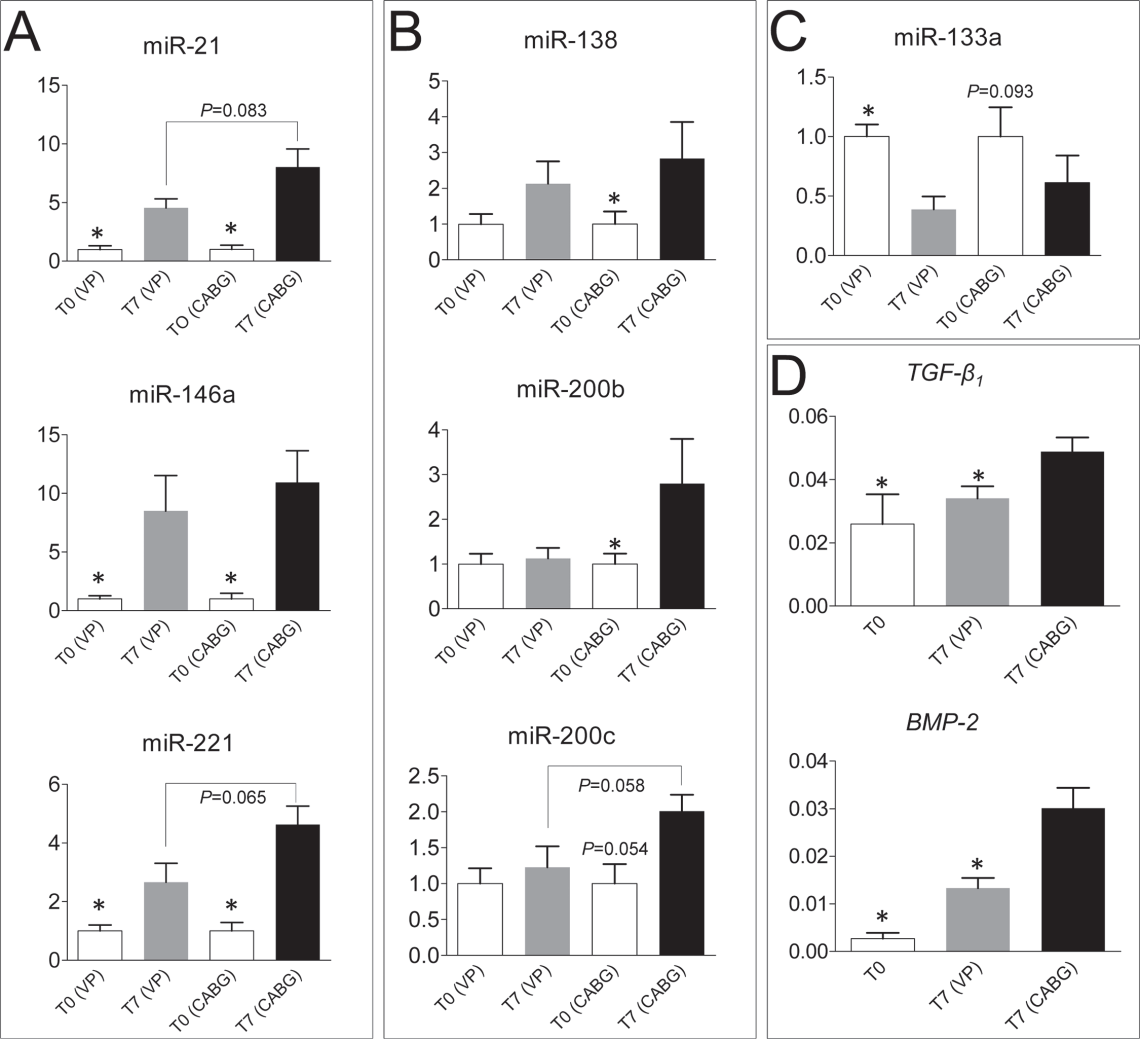
Assessment of biomechanical-dependent and -independent transcriptional regulation in the human SV. (A) q-RT-PCR experiment showing expression of miR-21/146a/221 in Native VP and CABG samples. Graphs show the fold changes observed in the expression of each micro-RNA (calculated by 2^-ΔΔCT^). Statistical comparisons were performed by paired/unpaired non-parametric t-test (Wilcoxon matched-pairs signed rank test; Mann-Whitney). * above control bars indicate a *P* < 0.05 value in the paired tests between treatments and controls, while numbers associated to connecting lines between bars indicate the *P* values achieved in the unpaired statistical tests adopted to compare the miRNAs expression in CABG *vs*. VP treatments (n = 8 for each treatment). (B) Expression of mechanically-regulated microRNAs (miR-138/200b/200c) in SVs exposed to CABG, but not VP condition. Data are expressed, grouped and statistically analyzed as in panel A. * above control bars indicate a *P* < 0.05 value (*P* = 0.054 for miR-200c) in the comparison between treatments and controls paired test, while the number associated to connecting lines between bars in the miR-200c graph indicates the *P* value rendered by the unpaired statistical test adopted to compare the miRNAs expression in CABG *vs*. VP treatments (n = 8 for each treatment). (C) miR-133a expression was reduced by the VP, but not CABG treatment. Data grouping, statistical comparison and level of significance as above (n = 8 for each treatment). (D) *TGF-β*_*1*_ and *BMP-2* expression are elevated in CABG but not in VP treated samples. Gene expression data are represented as relative expression (2^-ΔCT^) and were compared using one way ANOVA with Newman-Keuls post-hoc analysis. * above bars indicate a *P* < 0.05 value rendered by the statistical test (n = 4 Native; n = 7 VP; n = 9 CABG).

The upregulation of various micro-RNAs has been reported in vein graft failure models [17,18] and, more in general, cardiovascular diseases [40,41]. In order to assess whether culture of the SV in our *ex vivo* culture system recapitulates the miRNA-dependent pathology programming observed in previous studies [18], and to screen for biomechanical-specific gene expression activation, q-RT-PCR was performed on total RNA extracted from Native, VP- and CABG-conditioned veins. In our analysis, three differentially regulated miRNAs categories were found: *i)* miRNAs upregulated in VP and CABG veins (miR-21/146a/221; Fig. 6A); *ii)* miRNAs upregulated in CABG but not in VP treatment (miR-138/200b/200c; Fig. 6B) and *iii)* one miRNA (miR-133a), that was more pronouncedly downregulated by VP than by CABG pressure (Fig. 6C). Other tested miRNAs (miR-24/34a/126/145) were expressed in the vein at similar levels in all the tested conditions.

Various paracrine and chemotactic signaling pathways orchestrate vascular inflammation and intima hyperplasia in vein graft disease. This is the example of the Sonic Hedgehog (Shh)-dependent pathway, which directs SMCs proliferation in arterialized veins in mice [42], or that of the chemokine MCP-1, that promotes intimal migration of SMCs and cells with pericyte characteristics [43]. In addition, the observed increase in miR-21/200b/200c expression in CABG-stimulated samples suggested an implication of the TGF-β-dependent pathway [44,45]. In order to clarify whether mechanical stimulation is sufficient to elicit a paracrine signaling potentially involved in vessel pathologic progression and activation of adventitial progenitors, a q-RT-PCR was performed to determine the expression level of *Shh, MCP-1, TGF-β*_*1*_, *BMP-2* (a factor involved in SMCs calcification [46]), and *ZEB-1, SLUG* and *SNAIL* (transcription factors involved in endothelial-mesenchymal transition [47]). As shown in Fig. 6D, *TGF-β*_*1*_ and *BMP-2* were the only significantly modulated targets in CABG *vs*. VP and Native samples, while the other tested genes were unchanged or not expressed.

## Discussion

The multifactorial nature of vein graft disease makes particularly complicated the setup of effective treatments to prevent bypass restenosis in patients. The identification of cell and molecular pathways implicated in the disease has only in part allowed to circumvent the problem in clinics [48]. In fact, there are important biomechanical components that may primarily contribute to the pathology, which have been addressed only in part. The altered mechanical forces may act at very early post-implantation stages, when the vein segments become exposed to an increased shear stress determined by coronary arteries blood flow velocity, and to an increased wall strain consequent to the switch from a low and constant to a high and pulsatile pressure regimen [49]. While the effects of high and low shear stress on EC cells have been generally well characterized and may protect vessels from restenosis due to, e.g., enhanced Nitric Oxide production, the effects of the high pressure regimens may represent a primary damage signal promoting vascular remodelling from hours to days after arterialization. For these reasons, it is essential to address the specific contribution of the altered mechanical conditions in order to derive a pathophysiologic model of the disease, assess the biomechanical basis of vascular cells pathologic differentiation, or to uncover ‘trans-wall’ paracrine effects affecting the cell dynamics in the graft. In this regard, the conception of tools to accurately model the biomechanical forces in vein graft failure using human samples is an important step toward a comprehensive understanding of the disease at a molecular level, and setting up of novel translational interventions.

Despite the complications of vein graft occlusion manifest at relatively late stages after surgery, the disease process leading to bypass occlusion starts immediately after implantation, with a crucial contribution of mechanical forces [3]. For this reason, culturing SVs for a limited amount of time under appropriate biomechanical conditions has been considered sufficient to recapitulate some of the early events involved in the disease [50]. The *ex vivo* culture system adopted in the present study was used to expose segments of the human SV to a pulsatile pressure typical of the coronary circulation [26] adopting a relatively simple stimulation protocol for a period of 7 days. In keeping with previous reports [50], this system induced striking morphological changes in the cultured vessels. These were consistent with those reported in the vein conduits used as arterial grafts in patients [6,25], and included an around 45% reduction of the wall thickness and an equivalent increase in the luminal perimeter in CABG pressure stimulated veins (Fig. 2A-D). Interestingly, under arterial-like conditioning, the cross sectional area of the tissue was not affected (Fig. 2D) suggesting a reorganization of the extracellular matrix components, instead of a net tissue mass loss due to arterial-like wall strain. On the other hand, the significant decrease of the total nuclei density (Fig. 2E), mostly in circumferential SMCs layers, suggests that a major mechanical damage occurred mostly in the medial layer, although it was not associated to an elevation of apoptotic cells (Fig. 2F-G). This latter result, together with the increased presence of Ki-67^+^ cells in the wall of CABG-treated veins (Fig. 3A-B), confirms that our *ex vivo* culture system mimicked the early biomechanical effects on the human vein structural rearrangements occurring at early times after arterialization.

The adoption of a perfusion protocol consistent with the normal vein flow characteristics (the VP condition) enabled us to discriminate between changes occurring in the vein due to surgical manipulation *vs*. those attributable to pure biomechanical effects. For example, the analysis of MMPs activation did not reveal striking differences between the two conditions (Fig. 3C). This consolidates the notion that the vessel harvesting and manipulation procedures in the operating room cause unavoidable damages to the vessel wall that may reflect into its pathological programming. On the other hand, the remarkable difference in TIMP-1 protein expression between VP and CABG samples (Fig. 3D) suggests, for the first time, a direct effect of the arterial-like pressure on the suppression of remodeling protecting factors. Further studies to assess the control of TIMPs expression in mechanically stimulated SMCs are planned in our laboratory to address this specific point.

Increase in the *vasa vasorum* length density in the adventitia of veins grafts has been correlated to the sudden oxygen drop caused by the loss of blood supply at the time of vein excision from its natural bed [32]. The stimulation conditions (VP and CABG) adopted in the present study were set to avoid differences in the oxygen availability between the adventitial and the luminal surface of the veins. This prevented adventitial hypoxia to cause interferences in the biological responses of the SV tissue to the different pressure regimens during the culture period. For this reason, we hypothesize that the increase in the *vasa vasorum* length density observed in the CABG-stimulated veins (Fig. 4A) was a direct effect of the arterial-like biomechanical strain, likely activated by paracrine mechanisms.

An indication about a possible mechanism for strain-dependent activation of *vasa vasorum* cells comes from the results of the micro-RNAs and gene transcripts profiling in the vessels exposed to mechanical stimulation (Fig. 6) [17,18]. In particular, the finding of micro-RNAs (miR138/200b/200c/133a) specifically up or down modulated by arterial-like pressure indicates the existence of biomechanically-regulated transcriptional circuitries governed by mechanical strain possibly related to epithelium(endothelium)-mesenchymal transition [51], vascular stress [52] or inflammatory [53] responses. In keeping with this hypothesis, the search for putative targets of the miR-138/200b/200c signature in CABG-stimulated samples revealed, among others, a high probability of genes modulation functionally annotated in Notch, p53, HIF-1α, TGF-β and mTOR related pathways (S1 Table). Particularly interesting, in this regard, was the finding of a significant increase in the TGF-β1 mRNA expression in CABG-stimulated veins. In fact, apart from the reported effect of this factor on the differentiation of vascular SMCs [54], a recent study indicated a TGF-β1-dependent endothelial/mesenchyme transition as a novel mechanism of vein bypass stenosis either in animal vein arterialization models or in explanted vein bypass conduits from patients, where cells with mixed ECs/SMCs antigens expression were found in the intima [55]. Remarkably, in our study cells with a mixed endothelium/mesenchymal phenotype underneath the basal lamina was never observed, probably due to the limited culture time. On the other hand, the abundance of NG2^+^/CD44^+^/SM22α^+^ cells with pericytes (NG2^+^)/immature SMCs (SM22α^+^) characteristics at the boundary between the adventitia and the media, as well as and the appearance of Sox-10^+^/SM-MHC^-^ cells in the *vasa vasorum* of CABG-treated samples suggest that a paracrine signaling established by arterial-like pressure may drive *vasa vasorum*-derived cells transformation into progenitor-like cells with multipotential mesenchymal cells characteristics, potentially contributing to the pathology [10].

In summary, the present contribution indicates the existence of a biomechanical basis of the vein graft disease molecular programming in the human SV. In addition to consolidate observations up to date possible only in animal models [31], our bioengineering approach offers a valuable investigational tool to proceed with more refined human saphenous vein restenosis investigation, as well as to set up translational interventions aimed at minimizing the impact of vein bypass restenosis in the clinical setting.

## Materials and Methods

### Ethical statement

In the present investigation, the employment of human SV segments discarded after the end of bypass surgery interventions has been approved by the Ethical Committee at Centro Cardiologico Monzino, through the release of a written informed consent. It therefore conformed to the ethical standards laid down in the 1964 Declaration of Helsinki and its later amendments.

### SV segments preparation and culture

SV segments were supplied from the Department of Cardiovascular Surgery at Centro Cardiologico Monzino; these consisted in vein material discarded after the end of the CABG surgery (Table 1 for patients characteristics). The veins (harvested with a ‘no-touch’ method [4]) were stored at 4°C in Dulbecco Modified Eagle’s Medium (DMEM) supplemented with 10% Fetal Bovine Serum (FBS), 1% L-Glutamine, and 1% Penicillin/Streptomycin. The culture system was placed in a standard incubator at 37° C in a 5% CO_2_ atmosphere and for a culture period of 7 days. In both conditions, the culture system was filled with 42 ml of DMEM supplemented with 10% FBS, 1% L-Glutamine, and 1% Penicillin/Streptomycin. During the culture period, the medium was partially replaced at day 3. At day 7, SV segments were un-mounted from the *ex-vivo* culture system and were prepared for morphometric, immunofluorescence and biochemical/molecular studies. Before the culture, the excess vein tissue was harvested before mounting in the bioreactor a 5 cm vein segment. This strategy allowed to proceed with pairwise analysis of the CABG or VP stimulation *vs*. the Native conditions. After the culture, vessel segments were cut into small rings that were used to perform respectively, RNA, protein, histology, histochemistry and immunofluorescence, again using a pairwise analysis approach. Further details about the stimulation protocol are provided in the Results section.

**Table 1:** Patients principal characteristics.

Number of patients	Age (years; mean±SD)	Sex (%)	Diabetes(%)	Nicotin(%)	Hypertension(%)	Dyslipidemia(%)	Hyper-cholesterolemia (%)
28	67 ± 9	Male = 96	21	39	79	25	29
		Female = 4					

Saphenous vein grafts from 28 patients undergoing coronary bypass grafting were cultured either in VP or CABG conditions.

### Histology, immunohistochemistry and immunofluorescence analyses

To perform morphometric measures, sections were stained with Hematoxylin and Eosin (H&E) and Masson’s trichrome solution (Bio-Optica Milano SpA, Italy), according to manufacturer’s protocol. Six sections of each SV sample were acquired with a light microscope (Axiovert, Carl Zeiss, Germany) equipped with AxioVision Bio Software, at 10x magnification. For Ki-67 staining, 3 samples for each group were observed (6 sections for each sample). 3,3’-diaminobenzidine (DAB) substrate kit (SK-4100, Vector Laboratories) were used to reveal Ki67 staining. Briefly, slides were placed in 10 mM Sodium Citrate Buffer/0.05% Tween 20/buffer pH 6.0 and heated at 98°C for 10 minutes for antigen retrieval. Sections were then blocked with 3% Bovine Serum Albumin (BSA) in 0.1% Triton X-100. Blocking the endogenous peroxidase was performed by incubating sections in 3% H_2_O_2_/0.1% NaN_3_ for 10 minutes. Slides were incubated with primary Ki-67 antibody (1:100; Abcam, Cambridge, UK) for 1 hour at room temperature (RT) and subsequently incubated with biotinylated secondary antibody for 30 minutes at RT. Slides were then incubated with horseradish peroxidase for 30 minutes followed by incubation with DAB hydrochloride chromogen. TUNEL staining was performed on tissue sections using the DeadEnd Colorimetric TUNEL assay (Promega Italy). For both assays, sections were counterstained with hematoxylin. Immunofluorescence analysis for α-SMA (1:500 monoclonal mouse anti human, Dako), vWF (1:200 polyclonal rabbit anti human, Dako Cytomation), CD31 (1:200 polyclonal goat anti human, Santa Cruz Biotechnology) and CD34 (1:200 monoclonal mouse anti human, Dako) were carried out. These antigens were unmasked using a 10mM Tris-HCL/1mM EDTA/ buffer (pH 9.0) for 10 minutes under microwaves. After an incubation into a blocking solution for 1 hour at RT with 5% BSA sections were finally incubated over night at 4°C with primary antibodies in 3% BSA.

Staining with anti NG2 (1:50 monoclonal mouse anti human, Abcam Cambridge, UK 83508), CD44 (1:500 rat monoclonal anti human, Abcam Cambridge, UK), SM22α (1:500 polyclonal rabbit anti human, Abcam, Cambridge, UK), Sox-10 (1:200 monoclonal rat anti human, R&D, UK) and SM-MHC (1:200 goat polyclonal anti human, Santa Cruz Biotechnology) were performed after antigen unmasking by incubating sections for 20 minutes in Target Retrieval Solution (pH 6.0) (Dako) at 90°C, followed by blocking for 1 hour at RT with 5% BSA. Sections were then incubated over night at 4°C with primary antibodies in 3% BSA. After washing, sections were incubated with the following secondary antibodies: Alexa Fluor 488 anti-mouse, Alexa Fluor 546 anti-goat, Alexa Fluor 546 anti-rat and Alexa Fluor 633 anti-rabbit (1:200; Invitrogen, UK) for 1 hour at RT in 3% BSA. Nuclei were counterstained with DAPI for 15 minutes (Vector Laboratories, CA, USA). Sections were observed with a LSM710 confocal microscope equipped with ZEN2010D image acquisition/processing software (Carl Zeiss). Fields reported in the figures are representative of all examined fields.

### Morphometry methods and quantification of Ki-67^+^ and TUNEL^+^ cells

Morphometry of native and stimulated SV samples (wall thickness and luminal perimeter) was performed as previously reported [26] and according to Fig. 2A (n = 6). In addition, the cross sectional area of the wall tissue was estimated assuming a circular shape and using the measured wall thickness and luminal perimeter. The cross sectional area (*A*) was calculated according to:

A=w(πw+pinner)

where *w* is the wall thickness and *p*_*inner*_ is the inner perimeter. To evaluate the length density of the *vasa vasorum*, H&E stained sections of Native (n = 24), VP (n = 12), and CABG (n = 12) samples were analyzed. Digital images were acquired using a light microscope (Carl Zeiss, Germany) at a magnification of 40x. The complete image of each tissue section was reconstructed based on digital overlapping of pictures of the whole section. Using the AxioVision Bio Software (Carl Zeiss, Germany), the major and minor axes of vasa *vasorum* were measured. The classification of each vessel was performed based on its minor axis, which, for the definition of length density, represents the vessel diameter. *Vasa vasorum* in the 4–44μm range were subdivided into three subsets: small (4–14 μm), intermediate (14–24 μm), and large (24–44 μm) [33]. The *vasa vasorum* length density was calculated for each interval according to:

$$Ld=\sum_{i=1}^{n}R_{i}/Ad$$

for *n vasa vasorum* counted in a given adventitial area (*A*_*d*_), the length density (*L*_*d*_) corresponds to the sum of the ratios (*R*_*i*_) between the major and the minor axes of each *vasum vasorum*. In addition, *vasa vasorum* displaying no luminal region were not considered for morphometric determination of the length density. *A*_*d*_ was calculated on digital micrographs (2.5x) after manual contour identification using GIMP (GNU Image Manipulation Program, Version 2.6.12) and Image-J software (Version 1.47f-sofware for Java, National Institutes of Health, USA). A manual counting protocol of Ki-67^+^/TUNEL^+^ cells was performed on digital micrographs (20x) by using Image-J software (Version 1.47f-sofware for Java, National Institutes of Health, USA). The quantitative analysis of positive cells for Ki-67/TUNEL was accomplished by only one observer and in a blind fashion. The number of proliferating/apoptotic cells was normalized to the total cell count in the microscopic fields. In addition, the cell density was evaluated by normalizing the total cell count to the area of the tissue section. The area of the tissue section was calculated on digital micrographs (20x) using Image-J software.

### Protein methods

Zymographic analyses were performed on proteins extracted from Native, VP, and CABG samples. Frozen tissues were homogenized in Zymogram Buffer (50 mM TrisHCL/150 mM NaCl/1 μm ZnCl2/0.01% Triton X-100/2 mM EDTA/ buffer pH 7.4). Samples were centrifuged at 4°C, and the supernatant containing proteins was removed and quantified; 50 μg of extracted protein were mixed with zymogram loading buffer (62.5 mM Tris/HCl, pH 6.8, 10% glycerol, 2% SDS, 0.01% bromophenol blue) and run in 10% sodium-dodecyl sulfate–polyacrylamide gel electrophoresis (SDS-PAGE) containing 1 mg/ml type A porcine skin gelatin (SIGMA-Aldrich, Taufkirchen, Germany). To renature proteins, gels were washed twice in 2.5% Triton X-100 for 15 min at RT, and subsequently incubated in developing buffer (200 mM NaCl/50 mM Tris/20 mM CaCl2/ buffer pH 7.4) overnight at 37°C. Gels were stained with 0.5% Coomassie Blue in 45% methanol/10% acetic acid/45% water for 15 min, and de-stained until clear bands of lytic activity appeared. Image-J software was used for densitometric quantification. Data of Matrix Metallo-Proteinases (MMP)-2 were normalized to pro-MMP-2 as internal reference. MMP-9 quantification was not possible due to the proximity of the inactive and activated form of the enzymes in the gels.

Western Blotting analysis was performed on Native, VP, and CABG samples. Frozen samples were lysed in SDS Lysis Buffer (2% SDS/50 mM Tris/10% Glycerol/pH 6.8) and prepared for SDS–PAGE. Lysates were sonicated and boiled; the protein concentration was determined by Protein Assay kit (BioRad). 50 μg of extracted protein were loaded in a 10% SDS-PAGE followed by transferring onto a Polyvinylidene fluoride (PVDF) membrane and blocked for 1 hour at RT with 5% (w/v) milk in 0.1% PBS-Tween. Then the membrane was incubated in a primary antibody solution directed to α-SMA (1:5000 monoclonal mouse anti human, Dako), Tissue Inhibitor of Metallo-proteinases-1 (TIMP-1) (1:1000 monoclonal rabbit anti human, Cell Signaling Technology), and GAPDH (1:5000 polyclonal rabbit anti human, Santa Cruz Biotechnology). Incubation was followed by extensive washes in a 0.1% PBS-Tween buffer for 1 hour; membranes were incubated in anti-mouse (cat# 61–6520, Invitrogen, UK) or anti-rabbit (cat# 65–6120, Invitrogen, UK) horseradish peroxidase for 1 hour. Finally, membranes were washed in 0.1% PBS-Tween and then subjected to enhanced chemiluminescence detection (Amersham Life Sciences). Image-J software was used for densitometric quantification. α-SMA and TIMP-1expression levels were normalized to GAPDH as internal reference.

### MiRNA/gene expression analysis

For each miRNA (Table 2), quantitative real time polymerase chain reaction (qRT-PCR) was performed using miRNA Reverse Transcription Kit (Applied Biosystems, Foster City, CA) and the respective primers (miRNA TaqMan Expression assay, Applied Biosystems, Foster City, CA) according to manufacturer’s instructions, and using a 7900HT Fast Real-Time PCR System (Applied Biosystems, Foster City, CA). MiR-16 was adopted as normalization standard because of its persistent and stable expression throughout all considered samples regardless of the treatment. Relative miRNA expression levels were determined using the formula 2^-ΔCT^ (ΔCT = mean Ct [miRNA]—mean Ct [miRNA-16]). Fold increase was calculated using the formula 2^-ΔΔCT^ (ΔΔCT = ΔCT [treatment] – ΔCT [native]) [56]. For gene expression analysis, isolated total RNA integrity was verified by Agilent 7100 capillary electrophoresis system; samples with RNA Integrity Number greater than 6 were used for q-RT-PCR. 500 ng of total RNA was reverse transcribed with Omniscript Reverse Transcrition (Qiagen) in a volume of 20 μl, using 10 μM random hexamer primers (Roche), according to the manufacturers’ instructions. q-RT-PCR was carried out using SYBR green PCR kit (Applied Biosystem) and the Applied Biosystems 7900HT Fast Real-Time PCR System. Primers for GAPDH, β_2_-Microglobulin, TGF-β_1_, BMP-2, Slug, Snail-1, Shh and Zeb-1 were purchased from Integrated DNA Technologies, while primers for MCP-1 were custom-made and tested in house. Sequences and purchase numbers are shown in Table 2.

**Table 2:** List of genes and of reference sequence number in Native, VP- and CABG-stimulated veins.

Gene	IDT Reference #	RefSeq #
GAPDH	65558647	NM_002046
B_2_M	65558650	NM_004048
TGF-β_1_	65558671	NM_000660
BMP-2	65558677	NM_001200
Slug	65558662	NM_003068
Snai1	65558668	NM_005985
MCP-1	-	Fw 5’-ATAGCAGCCACCTTCATTCC-3’
		Rv 5’-ATCCTGAACCCACTTCTGCT-3’

### MiRNAs target prediction by functional gene annotation tools

Two different programs were used to perform prediction of functional pathways targeted by hsa-miR-138-5p, hsa-miR-200b-3p and hsa-miR-200c-3p: DIANA miRPath v.2.0 (http://diana.imis.athena-innovation.gr/DianaTools/index.php?r=mirpath/index) and miRSystem (http://mirsystem.cgm.ntu.edu.tw/index.php).

## Supporting Information

S1 Table Prediction of miR-138/200b/200c signature putative targets by functional annotation tools. (DOCX)

S1 Video The video shows the motion of the vessels during loading, stimulation and unloading phases of the CABG conditioning protocol represented in Fig. 1A. (MP4)

S2 Video The video shows the rotation around the vertical axis of the Z-stack 3D reconstruction of vasa vasorum in Native (T0) VP and CABG samples, as observed in SV transversal sections by confocal microscopy. EC cells are labeled by Red (CD31) and white (vWF) fluorescence, while SMCs are indicated by green (α-SMA).
(MP4)
