# Supplementary material for: Adventitial Vessel Growth and Progenitor Cells Activation in an Ex Vivo Culture System Mimicking Human Saphenous Vein Wall Strain after Coronary Artery Bypass Grafting
Source: PLoS One. 2015 Feb 17;10(2):e0117409. doi: 10.1371/journal.pone.0117409 (PMC4331547; doi:10.1371/journal.pone.0117409)
Supplement: S1 Table — (DOCX) [file pone.0117409.s001.docx]

**­Adventitial vessel growth and progenitor cells activation in an *ex vivo* culture system mimicking human saphenous vein wall strain after coronary artery bypass grafting**

Supplementary information

Supplementary Videos Legends

**Video S1.** The video shows the motion of the vessels during loading, stimulation and unloading phases of the CABG conditioning protocol represented in figure 1A.

**Video S2.** The video shows the rotation around the vertical axis of the Z-stack 3D reconstruction of *vasa vasorum* in Native (T0) VP and CABG samples, as observed in SV transversal sections by confocal microscopy. EC cells are labeled by Red (CD31) and white (vWF) fluorescence, while SMCs are indicated by green (α-SMA).

Supplementary Table

Table S1. Prediction of miR-138/200b/200c signature putative targets by functional annotation tools.

| Category | Term | Total Genes of the Term | Targets in the Term | Targets in Total Genes of the Term (%) | Targets in Total Targets of Submitted miRNA(s) (%) | Raw *P*  Value |
| --- | --- | --- | --- | --- | --- | --- |
| KEGG | Notch signaling pathway | 47 | 8 | 17,021 | 2,204 | 8,45127E-07 |
| PATHWAY_INTERACTION_DATABASE | HIF-1α transcription factor network | 65 | 9 | 13,846 | 2,479 | 1,05336E-06 |
| PATHWAY_INTERACTION_DATABASE | p53 pathway | 58 | 7 | 12,069 | 1,928 | 4,09764E-05 |
| PATHWAY_INTERACTION_DATABASE | Notch-mediated hes/hey network | 48 | 6 | 12,5 | 1,653 | 0,000119188 |
| KEGG | TGF signaling pathway | 84 | 7 | 8,333 | 1,928 | 0,000403412 |
| PATHWAY_INTERACTION_DATABASE | mTOR signaling_pathway | 68 | 6 | 8,824 | 1,653 | 0,000765858 |
| PATHWAY_INTERACTION_DATABASE | E2F transcription factor network | 73 | 6 | 8,219 | 1,653 | 0,00109732 |
| PATHWAY_INTERACTION_DATABASE | Notch signaling pathway | 59 | 4 | 6,78 | 1,102 | 0,0133913 |
